# Multi-Scale Fusion Underwater Image Enhancement Based on HSV Color Space Equalization

**DOI:** 10.3390/s25092850

**Published:** 2025-04-30

**Authors:** Jialiang Zhang, Haibing Su, Tao Zhang, Hu Tian, Bin Fan

**Affiliations:** 1 National Key Laboratory of Optical Field Manipulation Science and Technology, Chinese Academy of Sciences, Chengdu 610209, China; zhangjialiang@ioe.ac.cn (J.Z.); suhaibing@msn.com (H.S.); zhangtao226@mails.ucas.ac.cn (T.Z.); 2 Institute of Optics and Electronics, Chinese Academy of Sciences, Chengdu 610209, China; 3 School of Automation Engineering, University of Electronic Science & Technology of China, Chengdu 610209, China; 4 Sichuan Angzhi Weilai Technology Co., Ltd., Chengdu 610209, China; tigerwood.th@hotmail.com

**Keywords:** underwater image enhancement, multi-scale fusion, HSV color space

## Abstract

Meeting the escalating demand for high-quality underwater imagery poses a significant challenge due to light absorption and scattering in water, resulting in color distortion and reduced contrast. This study presents an innovative approach for enhancing underwater images, combining color correction, HSV color space equalization, and multi-scale fusion techniques. Initially, automatic contrast adjustment and improved white balance corrected color bias; this was followed by saturation and value equalization in the HSV space to enhance brightness and saturation. Gaussian and Laplacian pyramid methods extracted multi-scale features that were fused to augment image details and edges. Extensive subjective and objective evaluations compared our method with existing algorithms, demonstrating its superior performance in UCIQE (0.64368) and information entropy (7.8041) metrics. The proposed method effectively improves overall image quality, mitigates color bias, and enhances brightness and saturation.

## 1. Introduction

In recent years, spurred by global population growth and escalating terrestrial resource demands, there has been a remarkable focus on harnessing marine resources. The ocean, Earth’s largest ecosystem, stands as a repository teeming with diverse biological, energy, and mineral wealth. This heightened attention underscores the imperative of obtaining clear underwater imagery, pivotal across various domains including marine resource exploration, maintenance of underwater infrastructure, biodiversity studies, and seabed surveys. However, the underwater environment, characterized by light absorption and scattering in water [1], poses significant challenges. Consequently, underwater images often suffer from color shifts, diminished brightness, and weakened contrast, impairing the interpretability of vital information contained within.

Conventional image enhancement techniques like histogram equalization and contrast adjustments have shown limited efficacy in addressing underwater image quality issues. Recent research has spawned a variety of enhancement methods tailored specifically for underwater imagery. For example, He et al. [2]’s dark channel prior algorithm (DCP) leverages principles akin to fog removal to enhance visibility, yet it falls short in correcting the color biases inherent to underwater light attenuation. Building upon this, Drews et al. [3] refined the dark channel prior algorithm with the underwater dark channel prior algorithm (UDCP), focusing on blue and green channel processing but not fully addressing color distortions. Iqbal et al. [4] combined histogram and unsupervised color correction techniques to enhance contrast, though color fidelity remains a challenge. Zhang et al. [5] introduced the multi-scale Retinex algorithm (MSRCR) to the Lab color space, mitigating halo artifacts but with complex parameter adjustments. Garg et al. [6]’s adaptive histogram equalization technique effectively enhances contrast but overlooks color biases, while Ma et al. [7]’s approach improves brightness yet exhibits limitations.

The rise of deep learning presents a beacon of hope in the quest to rectify underwater image deterioration. This cutting-edge technology holds tremendous promise, offering sophisticated methodologies to tackle the myriad challenges posed by underwater environments. However, despite its potential, deep learning encounters hurdles in achieving broad applicability across diverse datasets. The crux of this challenge lies in the inherent complexity and variability of underwater scenes, which renders the task of developing models capable of generalizing effectively a daunting endeavor.

One of the primary impediments in leveraging deep learning for underwater image enhancement is the scarcity of paired training data. Unlike conventional image datasets, which are relatively abundant and readily available, acquiring paired data for underwater imagery is a formidable task. This scarcity stems from the logistical difficulties and high costs associated with collecting accurately labeled underwater image datasets. To address this, a large-scale real-world Underwater Image Enhancement Benchmark (UIEB) [8,9] was constructed, facilitating a comprehensive study and insightful analysis of underwater image enhancement algorithms. This benchmark allows for better training of CNNs, demonstrating enhanced visual quality in underwater images.

Moreover, deep learning algorithms often struggle to generalize across disparate underwater environments and conditions. The inherent variability in factors such as water clarity, depth, lighting conditions, and the presence of marine life poses formidable obstacles to achieving robust performance across diverse datasets. Models trained on specific underwater environments may falter when confronted with novel scenarios, highlighting the pressing need for enhanced robustness and adaptability.

Currently, multi-scale fusion emerges as a promising avenue in underwater image enhancement research. Ancuti et al. [10,11]. proposed a fusion method based on white balance, yielding sharpened and contrast-corrected images, and a multi-scale fusion strategy is adopted in the later study to avoid the sharp weight map transformation to produce artifacts in the low-frequency components of the reconstructed image. Gao et al. [12] introduced an enhancement method merging contrast-corrected and sharpened images for color restoration. Wu et al. [13] ventured into underwater image enhancement using multi-scale fusion generative adversarial networks. Liao et al. [14] proposes an underwater image enhancement method based on multi-task fusion, called MTF. The multi-scale fusion method is still a hot direction in the field of underwater image processing.

To tackle the degradation and low brightness plaguing underwater imagery while avoiding the influence on color, this paper proposes an innovative algorithm grounded in color correction and HSV color space [15] equalization, augmented by multi-scale fusion. Initial color restoration via an enhanced white balance method precedes HSV space equalization, enhancing brightness and saturation while mitigating color distortions. Subsequently, the multi-scale fusion technique amalgamates the images that have been enhanced for saturation and brightness, culminating in images exhibiting correct colors, crisp details, and heightened contrast and brightness. Through extensive comparison with contemporary underwater image enhancement algorithms, qualitative and quantitative evaluations underscore the efficacy of the proposed algorithm.

## 2. Materials and Methods

**Figure 1:**
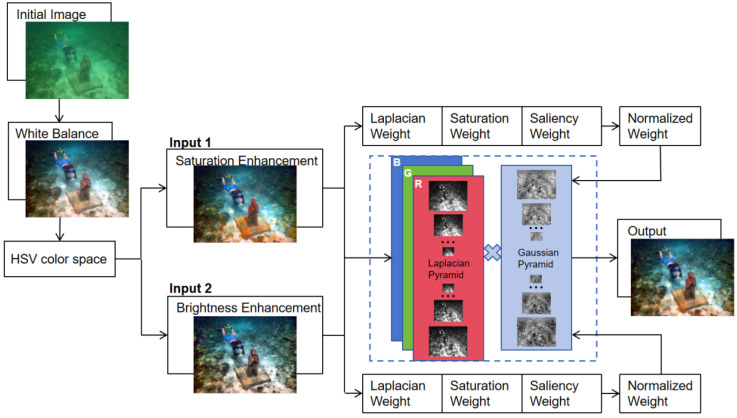
Flowchart of the proposed method.

Underwater image processing typically begins with color bias correction and enhancement of brightness and contrast. Traditional white balance methods, when dealing with underwater images that are mostly biased towards green and blue colors, can lead to insufficient brightness or over-enhancement of the red channel. Therefore, this paper employs an improved white balance method, which effectively corrects color biases across various types of images; due to absorption and scattering effects, underwater images suffer from reduced contrast and brightness, and traditional methods of enhancing these aspects in the RGB color space inevitably alter the color channels, undermining the effect of color bias correction. Consequently, in this paper, we perform equalization processing on the saturation and brightness channels separately within the HSV color space, which enhances saturation and brightness while avoiding impacts on the image’s color; based on the equalization in the HSV space, we have refined the weight calculation and fusion steps of the multi-scale fusion method, making image fusion more adapted to HSV space equalization, thereby further improving the image enhancement effects. The overall workflow of the algorithm proposed in this paper is shown in Figure 1:

The proposed underwater image enhancement algorithm consists of the following main steps:

1. Color balance using improved white balance
2. Brightness and saturation enhancement in HSV color space
3. Multi-scale fusion of enhanced omages

**Figure 2:**
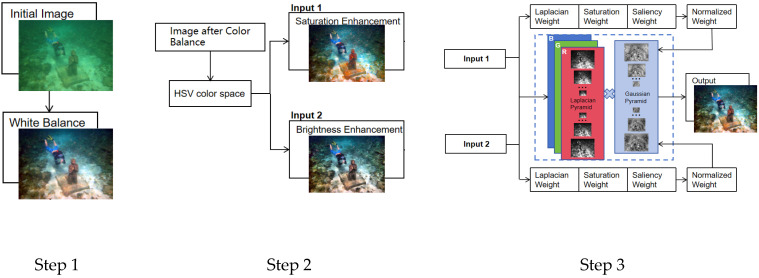
Three sub-‘algorithms’ of the ‘Algorithm’.

These steps are showed in Figure 2 and detailed in the subsections below.

### 2.1. Color Balance

The propagation characteristics of light in water significantly impact underwater photography and videography. Water molecules and suspended particles absorb and scatter light, often resulting in underwater images with color deviations and reduced contrast. Notably, light of different wavelengths attenuates at different rates in water. Short-wavelength blue-green light attenuates more slowly, whereas long-wavelength red light is almost completely absorbed at relatively shallow depths, such as 10 m. This phenomenon causes underwater images to typically exhibit a blue-green tint, while also significantly affecting the overall brightness and contrast levels. Traditional white balance techniques can effectively address color deviation issues by selecting a reference white point within the image to adjust the color balance, making the appearance of that point in the image more natural and accurate. However, when processing images at certain depths, these techniques can introduce new color distortions, often resulting in images that are overly corrected towards red after compensating for the blue-green tint.

This paper employs a white balance [16] method based on an improved assumption of the Gray World. The method begins with an in-depth analysis of the image’s color channels and then calculates an appropriate color adjustment ratio to reduce the overcompensation of long-wavelength light sources, such as red light. Simultaneously, by combining automatic contrast adjustment techniques based on histograms, the method further enhances the color richness and contrast of the image. This approach is highly effective for improving the overall color balance of underwater images.

Initially, the red (R), green (G), and blue (B) color channels are separated from the input RGB image.

(1) $$I_{RGB}=\{R,G,B\}$$

An affine transformation based on the cumulative histogram distribution is applied to the *R*, *G*, and *B* channels, assuming that the maximum values of the *R*, *G*, and *B* channels correspond to white and the minimum values correspond to black, with each channel’s maximum value range mapped to [0, 255].

(2) $$MAX=max({Avg}_{R},{Avg}_{G},{Avg}_{B})$$

The average luminance, ${Avg}_{c}(c\in \{R,G,B\}$), for each channel is computed, followed by calculating the ratio, Ratio = [Max/${Avg}_{R}$, Max/${Avg}_{G}$, Max/${Avg}_{B}$], of each channel’s maximum average luminance to the average luminance. This ratio is used to adjust each color channel. When performing the color balance method on pixels within each color channel, pixel values less than or equal to the bottom are adjusted to a saturation level equal to bottom, and values greater than or equal to top are adjusted to a saturation level equal to top. The histogram of each color channel is clipped by *N* pixels; hence, the bottom and top saturation levels are calculated using the cumulative histogram of the pixel values at the positions *N* × $S_{L}$ and *N* × (1 − $S_{L}$), respectively. Each channel is then adjusted separately, and the adjusted image pixel values are mapped to the [0, 255] range to fully utilize the entire grayscale range of the output image. The computation formula is as follows:

(3) $$f(x)=\frac{(x-bottom)\times 255}{top-bottom}$$

Let x be the input pixel value and $f\left(x\right)$ be the output pixel value, where ‘*bottom*’ and ‘*top*’ are defined as the lowest and highest saturation level pixel values, respectively. The formula for calculating the saturation level parameters $S_{L}$ is delineated as follows:

(4) $$S_{L}=0.005\times Ratio$$

**Figure 3:**
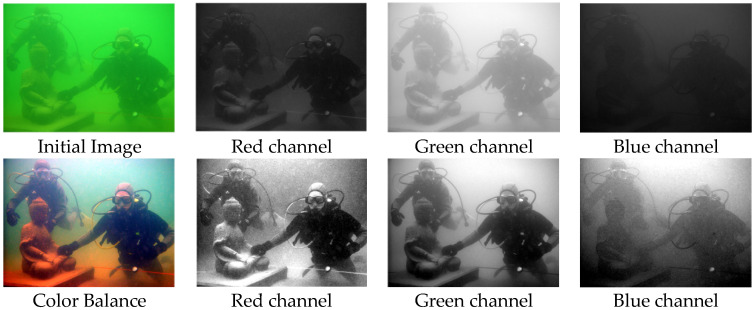
The original image and the color balanced image.

From Figure 3, it can be observed that the original image predominantly has a greenish tint, with the grayscale distribution being relatively uniform exclusively within the green channel among the RGB channels. Upon the application of the improved white balance process, the grayscale distribution across the red, green, and blue channels is notably more even when compared to the initial image, resulting in a clearer depiction of the image content. Consequently, the color balance method utilized in this study constitutes an effective approach for color correction in underwater imaging.

### 2.2. Saturation and Brightness Enhancement Based on HSV Color Space

Degradation of underwater images is not only due to color distortion caused by light attenuation but also due to a reduction in image brightness caused by backward scattering from suspended particles, resulting in decreased contrast. Reduced contrast affects image clarity, and contrast enhancement methods improve visibility by enhancing the image’s high-frequency information (detail). The human eye has a higher resolution for high-frequency information (details). Currently, there are various contrast enhancement methods, including those based on color space transformation, adaptive histogram equalization, and adaptive contrast enhancement techniques.

When processing color images, those captured in natural environments are subject to the influence of natural lighting, obstructions, and shadows, which means they are sensitive to changes in brightness. In the RGB color space, all three components are closely related to brightness, and any change in brightness affects all three components. However, this space does not intuitively separate color from brightness. Although the Lab color space separates brightness from color into independent channels, which prevents interference with color channels when enhancing brightness, it often results in brighter areas becoming even brighter and darker areas becoming darker, thus impacting brightness adjustment.

For these reasons, this paper employs the HSV (hue, saturation, value) color space, which more closely aligns with human color perception experience and provides an intuitive expression of color’s hue, saturation, and value. By decomposing the image into hue, saturation, and value channels, we can correct color bias, enhance saturation, and increase brightness separately, avoiding the interference between channels. This method is particularly important when processing underwater images, as it not only improves the overall visual effect of the image but also maintains the naturalness and accuracy of colors.

In the field of digital image processing, the HSV color model is a common way to represent color images, offering a color representation that aligns more with human visual perception than the hardware-based RGB (red, green, blue) color model. The HSV model describes the visual properties of color, including hue, saturation, and value. This model is particularly suited for image processing applications such as image segmentation, feature detection, and color filtering because it allows for more intuitive manipulation of color’s visual characteristics.

Hue: In the HSV model, hue represents the kind of color, measured in degrees, ranging from 0 to 360. Hue values represent color information, i.e., the position of the color in the spectrum, starting with red at 0 degrees, green at 120 degrees, and blue at 240 degrees. Complementary colors are determined by the position opposite on the color wheel: yellow (60 degrees), cyan (180 degrees), or magenta (300 degrees). Changes in hue reflect changes in color without involving lightness or saturation.

Saturation: Saturation describes the purity of the color, with values ranging from 0.0 to 1.0. Higher saturation means the color is more vivid, closer to pure spectrum colors. Lower saturation indicates the color is more washed out, closer to gray or white. In image processing, adjusting the saturation can enhance or weaken the intensity of the colors in the image.

Value: In the HSV color space, value represents the brightness of the color, with values ranging from 0.0 (completely black) to 1.0 (completely white). Value reflects the lightness or darkness of a color, independent of its hue and saturation. In image enhancement, adjusting the value is commonly used to improve the visual effect of images that are too dark or too bright.

In the context of image processing, ‘luminance’ and ‘value’ refer to different concepts, and it is crucial to use the correct term to avoid confusion.

Luminance: Luminance is a measure of the amount of light that is emitted or reflected by a surface. It is a photometric measure that takes into account the human eye’s sensitivity to different wavelengths of light. In color spaces like YUV or LAB, luminance (often represented as ‘Y’ or ‘L’) directly corresponds to the brightness of a pixel, factoring in its color information. Unlike value in the HSV color space, Luminance considers the contribution of each color channel (red, green, and blue) based on human perception, making it a more complex measure of brightness.

While both terms describe the brightness aspect of an image, they are calculated differently and used in different contexts. Luminance is often used in color spaces like YUV or LAB, where it represents a more accurate measure of brightness as perceived by humans. Value, however, is a simpler representation of brightness within the HSV color model, which is specifically designed for applications like image enhancement where adjusting the brightness independently of the color is required.

In summary, the HSV color model offers a way to represent colors in image processing that is in line with human visual perception. By separating different visual properties of colors, the HSV model enables more intuitive and effective color manipulation. Especially in various image processing tasks where color adjustment is needed, the HSV model demonstrates its unique advantages and application value.

In this paper, the color space of the image is converted from RGB to HSV:

(5) V=max(R,G,B)

(6) $$S=\left\{\begin{array}{c} 0\;\;\;if\;V=0\\ 1-\frac{min(R,G,B)}{V}\;\;otherwise \end{array}\right.$$

(7) $$H=\left\{\begin{array}{c} 0\;\;if\;S=0\\ \frac{60{}^{\circ}\times (G-B)}{S\times V}+360{}^{\circ}\;\;if\;V=R\\ \frac{60{}^{\circ}\times (B-R)}{S\times V}+120{}^{\circ}\;\;if\;V=G\\ \frac{60{}^{\circ}\times (R-G)}{S\times V}+240{}^{\circ}\;\;if\;V=B \end{array}\right.$$

(8) $$I_{HSV}=\{H,S,V\}$$

Then, histogram equalization is applied to the *S* (saturation) and *V* (value) channels while keeping the *H* (hue) channels constant. Finally, the image is converted back to the RGB color space to obtain an image with enhanced saturation and brightness. Assuming, in the HSV space, the grayscale image of the S channel is *S*(*x*, *y*), and the grayscale image of the V channel is *V*(*x*, *y*), the histogram equalization formula is as follows:

(9) $$S_{H}(x,y)=(\frac{255}{S_{max}-S_{min}})[S(x,y)-S_{min}]$$

(10) $$V_{H}(x,y)=(\frac{255}{V_{max}-V_{min}})[V(x,y)-V_{min}]$$

$S_{max}$ and $S_{min}$ respectively correspond to the maximum and minimum grayscale levels of the image *S*(*x*, *y*), while $V_{max}$ and $V_{min}$ respectively denote the maximum and minimum grayscale levels of the image *V*(*x*, *y*). $S_{H}(x,y)$ and $V_{H}(x,y)$ represent the grayscale images after histogram stretching. Following the histogram stretching in the HSV color space, the image is converted back into the RGB color space, yielding a contrast-enhanced image that enhances both the saturation and the brightness. The conversion formula is as follows:

(11) C=V×S

(12) $$X=C\times (1-|\frac{H}{60{}^{\circ}}\;\;mod2-1|)$$

(13) m=V−C

(14) $$(R,G,B)=\left\{\begin{array}{c} (C,X,0)\;\;if\;\;0{}^{\circ}\leq H<60{}^{\circ}\\ (X,C,0)\;\;\;if\;\;60{}^{\circ}\leq H<120{}^{\circ}\\ (0,C,X)\;\;if\;\;120{}^{\circ}\leq H<180{}^{\circ}\\ \;(0,X,C)\;\;if\;\;180{}^{\circ}\leq H<240{}^{\circ}\\ (X,0,C)\;\;if\;\;240{}^{\circ}\leq H<300{}^{\circ}\\ (C,0,X)\;\;if\;\;300{}^{\circ}\leq H<360{}^{\circ} \end{array}\right.$$

(15) (R,G,B)=(R+m,G+m,B+m)

**Figure 4:**
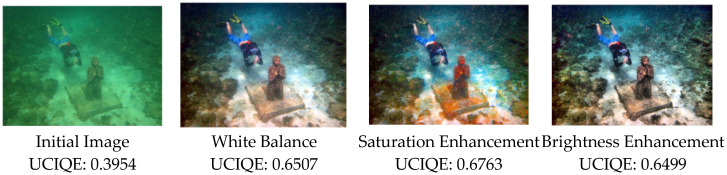
UCIQE at each step.

The saturation and brightness enhancement method presented in this paper is a rapid, adaptive approach to color image HSV histogram equalization, which is performed subsequent to white balance correction. Initially, color shifts are corrected using an improved white balance method, which is followed by the enhancement of saturation and brightness to further mitigate the effects of backscatter during the image formation process. The experimental results indicate that images enhanced with HSV saturation and brightness exhibit improvements in the underwater image quality evaluation metric UCIQE. Subjectively, images with enhanced saturation display richer colors, while those with enhanced brightness have more pronounced edges. Figure 4 displays the original input image, the image after white balance correction, the image after HSV saturation enhancement, and the image after HSV brightness enhancement.

### 2.3. Multi-Scale Fusion Technology

Following the improved white balance adjustment, images processed for saturation and brightness enhancement using the HSV histogram need to be fused to produce an enhanced image that retains their most significant features. Weights contain information and features of the image that are commonly utilized during the fusion process. In practice, no single weight map can represent all the important features of an image. This paper selects Laplacian weights, saturation weights, and saliency weights, which ensure that areas of the input image with higher contrast or brightness receive higher values. To better describe the spatial relationships between degraded regions, the weights are designed in pixel form.

Laplacian Weight: The Laplacian weight measures the global contrast information of an image, assigning high values to regions with edges and texture. This weight is calculated from the absolute value of the Laplacian filter applied to the luminance channel, as shown below.

(16) $$W_{L}(x)=∥L^{Lap}(x)∥$$

where *x* represents the pixels of the image and $L^{Lap}(x)$ denotes the luminance channel values after filtering with a Laplacian filter. The Laplacian weight is responsible for highlighting areas with high-intensity variations. However, this weight alone is insufficient for restoring local contrast in flat areas. Therefore, two additional weights are employed during the fusion process: the saturation weight and the saliency weight.

Saturation Weight: The primary objective of this weight is to enhance the vividness of colors in underwater images, making them more attractive and legible. To obtain this weight, the standard deviation between the input luminance and each color channel is calculated at every pixel location. This paper improves upon the traditional method for calculating saturation weight, aiming to evaluate and reinforce the visual impact of color richness in images. Unlike conventional saturation analysis methods, this weight calculation does not solely focus on the purity of colors but also considers the contribution of color distribution and intensity to the overall visual perception. By transitioning to the HSV color space and focusing on the saturation channel, this method effectively identifies image regions that are rich in color and visually significant. The formula for weight calculation is the following:

(17) $$W_{S}(x)=\sqrt{1/3{\left((R(x)-S(x)\right)}^{2}+{\left((G(x)-S(x)\right)}^{2}+{\left((B(x)-S(x)\right)}^{2}}$$

In the formula, *R*(*x*), *G*(*x*), and *B*(*x*) represent the values of the RGB color channels, while *S*(*x*) is the saturation channel value in the HSV space. By combining the RGB color space with the saturation channel of the HSV space, regions with high saturation are assigned higher values, while other areas are given lower values.

Saliency Weight: This weight is designed to differentiate between key areas of the image and their adjacent regions, thereby effectively highlighting objects in underwater environments that may be difficult to detect. The core principle of this type of weight is to identify and emphasize those image regions that contain rich information. The goal of saliency detection is to identify areas that exhibit significant visual differences from their surroundings, such as in color, brightness, texture, and the like. These areas are usually the parts of an image that most attract the observer’s visual attention and are therefore especially important in visual processing. The main purpose of this paper is to highlight the regions between high and low brightness in the images, and the formula for weight calculation is the following:

(18) $$W_{Sa}(x)=∥I^{whc}(x)-I^{a}∥$$

In the expression, $I^{a}$ represents the luminance channel of the input image, and a is the index of the input image; $I^{whc}(x)$ denotes the result after low-pass filtering within the neighborhood.

Normalized Weights: To produce consistent results, the three weight values $W_{L}(x)$, $W_{S}(x)$, and $W_{Sa}(x)$ are combined into normalized weights for each input image. For each input, the normalized weight $\bar{W_{j}}(x)$ is calculated as follows:

(19) $$\bar{W_{j}}(x)=\sum_{i=1}^{3}W_{j}^{i}/\sum_{j=1}^{2}\sum_{i=1}^{3}W_{j}^{i}$$

where *i* represents the index of the three weights, and *j* denotes the index of the input image. The single image fusion method for underwater images can result in severe halo artifacts. This paper employs Gaussian and Laplacian pyramid algorithms to decompose the images and then uses a multiscale fusion method to merge the decomposed images.

The straightforward method of linearly superimposing images enhanced by disparate techniques could induce artifacts and halos in the resultant imagery. This manuscript proposes the application of a multiscale fusion algorithm for further image enhancement, in which images, post-enhancement, and their normalized weight maps are subjected to decomposition via Laplacian and Gaussian pyramids, followed by a multiscale fusion process.

In the realm of digital image processing, multiscale image fusion algorithms have garnered considerable interest for their exemplary performance across a multitude of applications. To actualize the efficacious fusion of color correction and HSV color space equalization techniques, and to further augment image quality via a multiscale approach, we advocate for the construction of Gaussian and Laplacian pyramids. These pyramid constructs are formulated atop images post color correction and equalization processing, ensuring fidelity in color and the naturalness of visual effects throughout the enhancement procedure.

Gaussian Pyramid Construction: Fundamental to the multiscale fusion algorithm, the Gaussian pyramid furnishes the requisite input for the ensuing Laplacian pyramid. Through a systematic decrement in image resolution, the Gaussian pyramid yields imagery at diverse scales, tantamount to scrutinizing the images post color correction and luminance equalization across varying observational scales, ensuring comprehensive information capture from micro details to the macro scale.

Laplacian Pyramid Construction: An advancement of the Gaussian pyramid, the Laplacian pyramid is geared towards the capture of an image’s minute details, a critical element in preserving crisp edges and particulars within the terminally fused enhanced image. This procedure is contingent on antecedent color correction and luminance equalization, for it is post these adjustments that details are most authentically and effectively apprehended.

Fusion Weights: Post color correction and HSV color space equalization, weights are ascertained to assure that during the multiscale fusion, the selection of pertinent information is governed by the content’s significance and quality within the imagery. This junction serves as the pivotal integration of the techniques proposed herein with the multiscale fusion strategy, ensuring the augmented image manifests a harmonious balance of color, luminance, and contrast.

Image Reconstruction: The culmination lies in amassing all information procured from antecedent methodologies to compile the ultimate enhanced image, denoted as *F*(*x*). Commencing from the most rudimentary scale and methodically amalgamating data from each successive layer, the enhanced image not only experiences an elevation in local detail but also achieves a more cohesive overall visual effect. The triumph of this process is ascribed to the foundational color correction and HSV color space equalization, as well as to the scrupulously devised multiscale fusion strategy. The formula for image fusion reconstruction is delineated as the following:

(20) $$F(x)=\sum_{l=1}^{5}\sum_{j=1}^{2}G_{l}\{\bar{W_{j}}(x)\}L_{l}\{I^{j}(x)\}$$

The variable *l* represents the number of levels in the pyramid, and *j* denotes the index of the input image, with *l* = 5 and *j* = 2. $L_{l}$ stands for Laplacian pyramid decomposition, while $G_{l}\{\bar{W_{j}}\}$ represents Gaussian pyramid decomposition. Through multiscale image fusion, the algorithm is capable of reconstructing a complete image that preserves detail information across all scales. The primary advantage of the multiscale image fusion algorithm lies in its ability to effectively combine the strengths of different images, resulting in a visually more appealing and information-rich composite image. This type of algorithm has wide applications in various fields, such as underwater image processing, satellite imagery, medical imaging, and security surveillance.

**Figure 5:**
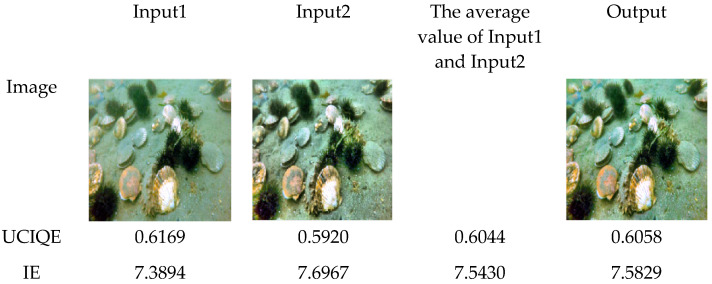
UCIQE and IE of Input1, Input2, The average value of Input1 and Input2, Output.

It can be seen from Figure 5 that the UCIQE of image Input1 after saturation enhancement is higher than the UCIQE of image Input2 after brightness enhancement, while the IE of Input2 is lower than that of Input. The UCIQE and IE of the image output obtained by multi-scale fusion are more balanced and comprehensive, which are slightly higher than the average values of Input1 and Input2.

## 3. Results

### 3.1. Objective Evaluation Without Reference

Underwater imagery often lacks corresponding true undegraded images, making it challenging to evaluate the quality of such images with reference-based metrics. Consequently, no-reference metrics are commonly utilized for assessing underwater image quality. Three widely recognized measures employed for this purpose are the Underwater Color Image Quality Evaluation (UCIQE) [17], the Underwater Image Quality Measure (UIQM) [18], and the Image Entropy (IE). Each of these metrics offers a unique perspective on assessing the overall perceived quality of underwater images, taking into account the contribution of various attributes to the overall image quality. This paper employs these indices to evaluate the performance of the proposed algorithm. The definitions related to the evaluation metrics are as follows:

- Underwater Color Image Quality Evaluation (UCIQE): UCIQE is a no-reference image quality metric specifically designed for underwater images, proposed by Yang et al. Unlike traditional quality metrics, UCIQE does not require a reference image for comparison. It assesses image quality based on the chromaticity, contrast, and sharpness of underwater images. This metric is a linear combination of three independent components: the standard deviation of chroma, the contrast of luminance, and the average of saturation. The index was initially developed for images from underwater pipeline inspections, with coefficients for different weights being derived through fitting. UCIQE has been widely adopted due to its effectiveness in reflecting color distortions and blurriness commonly encountered in underwater imaging.

$$UCIQE=c_{1}*\delta_{c}+c_{2}*{con}_{l}+c_{3}*\mu_{s}$$

In the expression above, $\delta_{c}$ is the standard deviation of color concentration, ${con}_{l}$ is the luminance contrast, and $\mu_{s}$ is the average saturation. $c_{1},c_{2},c_{3}$ are weighted coefficients with values of 0.4680, 0.2745, and 0.2576, respectively. These coefficients are derived from data in reference. A higher UCIQE value indicates better image quality.

- Underwater Image Quality Measure (UIQM): UIQM is another no-reference image quality assessment metric for underwater images, proposed by Panetta et al. It evaluates image quality based on three key aspects: colorfulness, sharpness, and contrast. Specifically, UIQM comprises a colorfulness measure that assesses deviations in the image colors from the typical blueish tint of underwater images; a sharpness measure that quantifies the amount of discernible detail within the image; and a contrast measure that evaluates the degree of visibility in the image. These components are combined to provide a comprehensive assessment of underwater image quality.

$$\mathrm{UIQM}=c_{1}\mathrm{UICM}+c_{1}\mathrm{UISM}+c_{1}\mathrm{UIConM}$$

UICM represents the colorfulness measure of the image; UISM denotes the sharpness measure of the image; and UIConM is the image contrast measure; with weighted coefficients of 0.0282, 0.2953, and 3.5753, respectively. A higher UIQM value signifies better quality of underwater images.

- Image Entropy (IE): Image entropy is a statistical measure used to describe the texture of an image. In the context of underwater imagery, a high entropy value typically indicates a richness of detail and texture, suggesting that the image is of high quality and contains a substantial amount of information. Conversely, a lower entropy value usually signifies a smoother image with a lack of details, which might be due to blur, low contrast, or color degradation. Although IE is a more general metric and not specifically designed for underwater images, it offers valuable insights into the degree of detail preserved during the image enhancement process.

$$IE=-\sum_{i=1}^{256}\left(p(i)*{log}_{2}(p(i))\right)$$

Let p(i) denote the probability of occurrence for each grayscale value in an image. A larger value of IE suggests that the image contains a higher quantity of information, which is indicative of better image quality.

To further validate the enhancement effects of the proposed method on underwater images and to verify the performance of the algorithm presented in this paper, we selected UDCP, Ancuti, Retinex, and L^2UWE [19] algorithms for comparison. These methods were compared using both subjective and objective metrics, including color restoration, detail contrast, UCIQE, UIQM, and information entropy. The experimental environment was Matlab R2022a, running on a computer equipped with an Intel Core i7-12700H CPU, 16 GB of RAM, and a base clock speed of 2.3 GHz. In this paper, images with different degradation levels are processed respectively, and the specific experimental results are shown in Figure 4.

**Figure 6:**
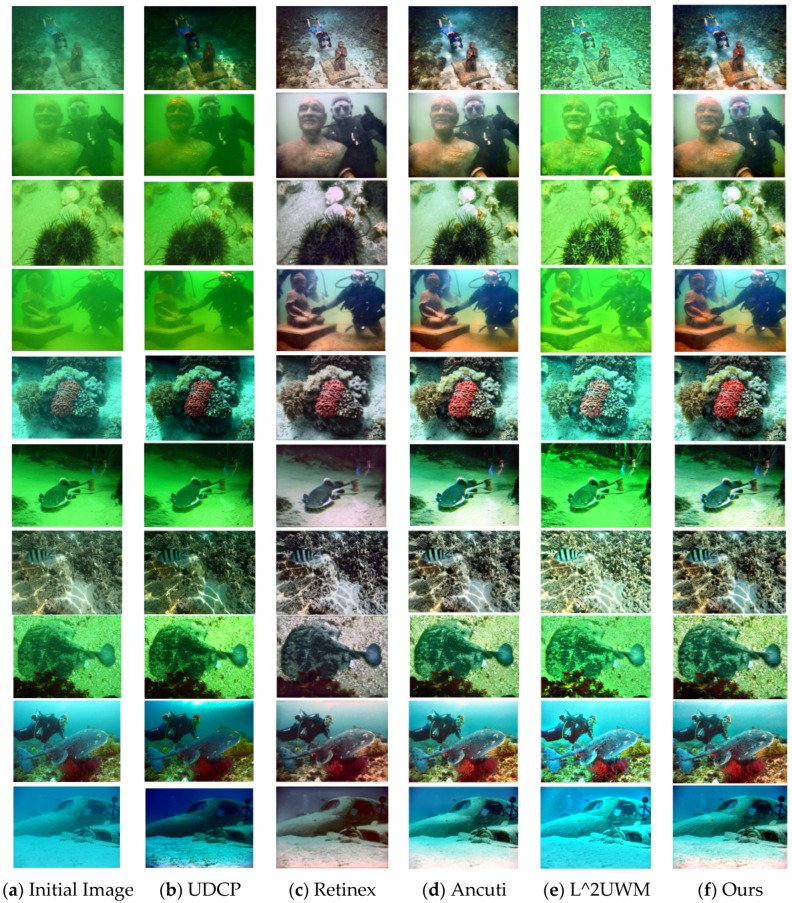
Comparison on challenging underwater scenes, from left to right: (**a**) Initial image, (**b**) UDCP method, (**c**) Retinex method, (**d**) Ancuti method, (**e**) L^2UWM method, and (**f**) our enhancement method.

As illustrated in Figure 6, the selected algorithm’s performance across various scenes and lighting conditions is showcased. The algorithm proposed in this paper achieves outstanding results in the majority of scenarios. Subjectively speaking, images processed by the algorithm presented in this document appear clearer and sharper with richer colors compared to those processed by other algorithms, maintaining good performance across different color biases and lighting conditions.

From an objective standpoint, the test results are presented in Table 1, Table 2 and Table 3. Comparatively, the algorithm proposed in this paper surpasses other algorithms on the UCIQE and IE metrics, demonstrating that the images processed by our method possess higher image quality and information content. On the UIQM metric, it performs slightly worse than the Retinex algorithm, mainly due to deficiencies in color bias correction within our algorithm. Specifically, when processing images with a blue bias, there is a tendency for an excessive enhancement of red tones, and UIQM includes a colorfulness measure to evaluate the deviation of colors in the image from the typical blue hue of underwater imagery.

### 3.2. SIFT

The primary goal of image enhancement techniques transcends mere visual improvement; its deeper significance lies in laying a robust groundwork for various intricate high-level computer vision tasks. Among these tasks, feature point matching stands as an indisputably fundamental and pivotal aspect, serving as the backbone for advanced visual applications like object recognition, image stitching, and three-dimensional modeling. Thus, evaluating the feature point matching performance of images processed through enhancement algorithms emerges as a crucial metric for assessing their efficacy.

**Figure 7:**
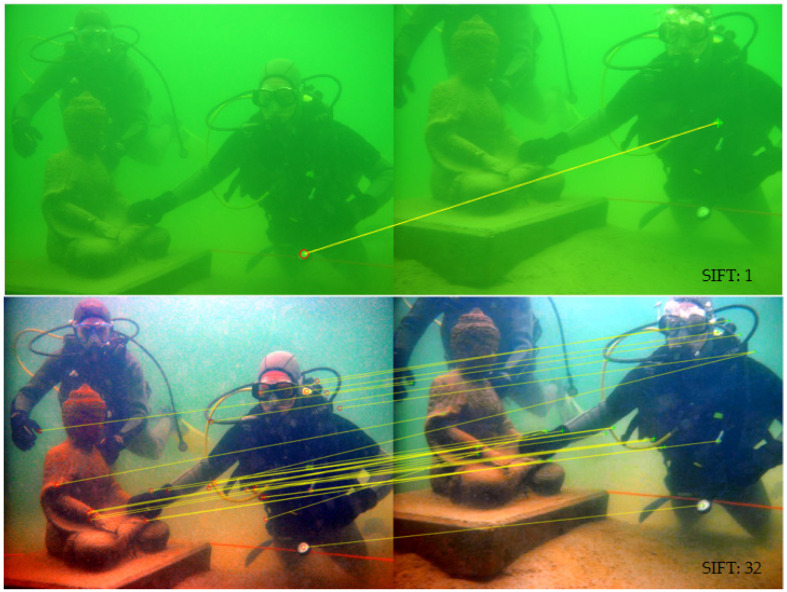
SIFT matching point test.

In the validation process of image enhancement algorithms, the Scale-Invariant Feature Transform (SIFT) [20] matching point test offers a dependable method for quantitatively analyzing enhancement effects. The SIFT algorithm adeptly identifies and characterizes key points within images and their surrounding areas, facilitating the precise matching of these feature points across disparate images. This ensures that the enhancement operations conducted by the algorithm significantly enhance the quality and precision of subsequent vision tasks. The figure below illustrates a comparative outcome of the SIFT feature point matching test pre and post application of image enhancement. This comparison not only delineates the alteration in the number of matching points but also underscores the potential influence of the algorithm’s enhancement on the detection and matching capabilities of feature points. The result of SIFT matching point test is showed in Figure 7:

Results from the SIFT matching point test indicate that the original image had only a single pair of matching points, whereas the image enhanced by the algorithm proposed in this paper exhibited a substantial increase to 32 pairs of matching points. This significant improvement in the number of matching points enhances the image’s feature representation capability, thereby benefiting subsequent computer vision tasks. The enhanced feature representation not only facilitates more accurate recognition and tracking but also improves the robustness of vision-based applications in varying conditions, laying a stronger foundation for reliable image analysis and interpretation.

## 4. Conclusions

Underwater environments present challenges such as color distortion, low contrast, and loss of detail in imagery. This paper introduces an innovative underwater image enhancement algorithm that combines adaptive equalization in the HSV color space with a multi-scale fusion approach, offering a significant improvement over existing techniques.

Our method starts with an improved white balance technique to address underwater color distortion, which is followed by brightness and saturation enhancement using adaptive equalization in the HSV color space. This ensures that the brightness and saturation are improved without affecting the color. Another key innovation is the multi-scale fusion using Gaussian and Laplacian pyramids, with modified normalization weights tailored for the HSV color space, enhancing image details and edges.

Assessments show that our algorithm significantly improves color correction, detail enhancement, and overall contrast and brightness. It outperforms other algorithms in UCIQE and IE metrics and shows better performance in SIFT matching point tests. However, it has limitations in color bias correction, underperforming in UIQM assessments and producing oversaturated results in blue-biased environments.

Future work will focus on optimizing color bias correction before HSV equalization and exploring advanced deep learning techniques to enhance robustness and applicability across various underwater environments.

## Figures and Tables

**Table 1:** UCIQE of different underwater images processed by different methods.

Image	Initial Image	UDCP	Retinex	Ancuti	L^2UWM	Ours
Image1	0.4355	0.2589	0.5730	0.6421	0.2589	**0.6670**
Image2	0.4364	0.2589	0.5723	0.6278	0.2593	**0.6499**
Image3	0.5240	0.2587	0.5395	0.5720	0.2591	**0.5913**
Image4	0.3954	0.2596	0.6431	0.6842	0.5328	**0.6941**
Image5	0.5133	0.2587	0.5871	0.6366	0.2587	**0.6551**
Image6	0.5667	0.2587	0.5612	0.5949	0.2590	**0.6071**
Image7	0.5403	0.2587	0.5624	0.5950	0.2587	**0.6247**
Image8	0.5679	0.2587	0.5825	0.6224	0.2598	**0.6370**
Image9	0.6482	0.2603	0.6337	0.6738	0.2595	**0.6808**
Image10	0.4420	0.2587	0.5878	0.6237	0.2587	**0.6298**
Avg	0.50697	0.25899	0.58426	0.62725	0.28645	**0.64368**

**Table 2:** UIQM of different underwater images processed by different methods.

Image	Initial Image	UDCP	Retinex	Ancuti	L^2UWM	Ours
Image1	1.5692	0.6144	**4.6181**	4.3992	3.3578	4.4556
Image2	0.8171	0.9112	**4.3584**	4.1792	1.1468	4.1768
Image3	2.4891	1.5223	**5.1402**	3.7155	3.9046	3.5338
Image4	0.0086	0.5537	**4.5593**	4.2175	0.6246	4.2209
Image5	2.7350	2.9442	**5.3241**	4.5343	4.3154	4.0058
Image6	1.6510	1.4530	**4.7705**	3.3892	1.7610	2.9623
Image7	4.1433	−0.00016569	**4.8622**	4.6475	0.0282	4.3389
Image8	2.7437	3.2210	**5.5676**	3.8919	5.5341	3.6389
Image9	2.7501	2.8431	4.7506	3.6544	**11.2107**	3.5938
Image10	−0.6786	1.0072	**3.8712**	1.8699	0.5997	1.5851
Avg	1.82285	1.506993431	**4.78222**	3.84986	3.24829	3.65119

**Table 3:** IE of different underwater images processed by different methods.

Image	Initial Image	UDCP	Retinex	Ancuti	L^2UWM	Ours
Image1	6.7556	0.4050	7.6409	**7.7450**	0.7084	7.7318
Image2	7.0016	0.9997	7.6775	7.8622	0.3147	**7.8698**
Image3	7.2909	0.9197	7.6827	7.7164	0.7028	**7.8050**
Image4	6.3964	0.7417	7.6231	7.6266	0.0134	**7.6443**
Image5	7.3428	0.8465	7.6257	7.8335	0.9118	**7.8600**
Image6	7.3689	0.9980	7.5956	7.8161	0.8324	**7.9088**
Image7	7.4718	0.6450	7.6876	7.8332	0.9098	**7.8382**
Image8	7.6236	0.9818	7.6648	7.8708	0.8515	**7.9285**
Image9	7.6105	0.7244	7.6817	7.7538	0.8963	**7.7984**
Image10	6.5511	0.9238	7.6498	7.6057	0.4505	**7.6562**
Avg	7.14132	0.81856	7.65294	7.76633	0.65916	**7.8041**

## Data Availability

Data is unavailable due to privacy or ethical restrictions.
